# Psychological Flexibility Profiles and Mental Health Among University Students with Left-Behind Experience: A Latent Profile Analysis

**DOI:** 10.1007/s10578-024-01720-3

**Published:** 2024-06-12

**Authors:** Meng Ning, Qirong Chen, Yamin Li, Chongmei Huang

**Affiliations:** 1 Clinical Nursing Teaching and Research Section, The Second Xiangya Hospital of Central South University, 139 Renming Middle Road of Furong District, Changsha, 410000 Hunan China; 2 School of Nursing at Ningxia Medical University, 1160 Shengli Street of Xingqing District, Yinchuan, 750101 China; 3 Xiangya School of Nursing, Central South University, Changsha, Hunan China

**Keywords:** Left-behind; Psychological flexibility; Mental health; Latent profile analysis

## Abstract

The left-behind experience as an adverse childhood experience has a negative impact on the mental health of university students. Psychological inflexibility mediated the relationship between adverse childhood experiences and mental disorders, but no similar findings were drawn in psychological flexibility (PF). More research is needed to understand the relationship between PF and mental health of university students with left-behind experience. To investigate the relationship between PF profiles and mental health and sleep quality of university students with left-behind experience based on latent profile analysis. A sample of 1988 Chinese university students with left-behind experience were recruited to complete an online survey. Participants provided demographic information and completed validated measures of PF and mental health. Latent profile analysis was used to identify patterns of PF, and logistic regression analysis was used to examine the relationships among these variables. We found four PF profiles among participants, with the largest number being the moderately flexible profile (n = 808, 40.6%). The level of PF was positively correlated with mental health and sleep quality (all *P* < 0.001). Females, being left behind at a young age and for a long time, and having little contact with parents were associated with low PF (all *P* < 0.05). Our study highlights the importance of focusing on the PF of university students with left-behind experience and left-behind children, and the need for interventions to improve their PF and thus their mental health.

**Supplementary Information:**

The online version contains supplementary material available at 10.1007/s10578-024-01720-3.

## Introduction

Mental disorders contribute to nearly one-fifth of the global disease burden, and its associated annual global cost may reach US$6 trillion by 2030 [6]. The university period is a critical transition from late adolescence to adulthood [2, 9], with physical function (brain transition) and psychosocial environment (role and social support) changing. As a result, university students are more likely to suffer from mental disorders and the average age of the first onset of mental disorders is 21 [47]. Mental disorders among university students are related to health, academics, career, and intimate relationships [24, 52].

Among the risk factors for mental disorders among university students, the left-behind experience is one of the identified risk factors [14, 26]. The (being) left-behind experience was defined as the experience of a child who remained in their place of origin when one or both of their parents left to work for six months or more [45]. Up to 2020, millions of children worldwide have been left behind, especially in low-income or middle-income countries like Philippines, Kyrgyzstan and China. As labour migration increases globally, the number of left-behind children continues to grow [46]. Different left-behind characteristics have different impact on mental health, for example, the absence of the mother is more likely to cause psychological problems and self-injurious behavior than the absence of the father, and separation at young ages has a higher risk for mental health disorders than separation in adolescence [26, 46]. University students with left-behind experience are more likely to suffer from mental health problems such as anxiety, depression, obsession, and substance abuse than those who have not been left behind [27]. Therefore, it is necessary to explore the relation between different left-behind characteristics and mental health problems and find risk and protective factors for mental health among university students with left-behind experience.

Some evidence supports that psychological inflexibility mediates the relation between adverse childhood experiences and mental disorders [25, 30, 39], but no similar findings for psychological flexibility (PF). PF refers to “the ability to contact the present moment more fully as a conscious human being, and to change or persist in behavior when doing so serves valued ends” [21]. PF includes three core components associated with mental health: values, acceptance and cognitive defusion. Living a valued life is critical to good mental health [42]. Acceptance and cognitive defusion are two basic psychological processes, and low levels of acceptance and cognitive defusion are associated with mental disorders [3, 41]. Evidence suggests that PF is associated with psychological functioning and mental health [8]. Studies focused on PF of university students indicated that a high level of PF is related to better mental health [4, 35]. However, little evidence examined the relation between PF and mental health among university students with left-behind experience.

Current studies examining the relation between PF and mental health have mostly used a single component of PF [34, 36] or a total PF score [13] to represent the level of PF. However, a single component cannot fully represent PF [51], and there are some problems with using a total PF score. First, the total score approach assumes that each component of PF affects mental health to the same degree. However, different components have different emphases, such that values progress is more related to life satisfaction [40] and acceptance and cognitive fusion are more related to mental disorders [11]. Second, the total score approach ignores the heterogeneity of the study population, yet individuals typically perform inconsistently on different components of PF [10]. The lack of heterogeneity hindered the mechanistic elucidation of the relation between PF and mental health and the concretization of interventions. Therefore, more appropriate measures of PF levels need to be explored to guide interventions. In recent years, person-oriented methods, such as latent profile analysis (LPA) [22], have been applied to identify different patterns of PF [4, 43]. However, the relation between the different PF profiles and the mental health and left-behind characteristics of university students has not been fully studied. Understanding the impact of profiles on mental health based on different components of PF can help identify at-risk university students with left-behind experience, develop specific interventions to improve PF, and ultimately improve the mental health of this group.

To bridge the knowledge gap, the current study investigated the relation between PF profiles and mental health and sleep quality of university students with left-behind experience based on latent profile analysis. We aimed to (1) analyze the potential profiles of PF of university students with left-behind experience; and (2) explore the relation between the profiles of PF and mental health and sleep quality and (3) explore the association between left-behind experience characteristics and PF.

## Methods

### Study Design

The study was an anonymous online cross-sectional survey involving university students with left-behind experience, which was conducted between October and December 2021, in China.

### Procedure and Participants

Convenience and snowballing approaches were used to recruit participants in this study. Through their networks, the research team members sent online recruitment advertisements to potential participants via social media (WeChat and QQ). The advertisement included detailing inclusion criteria, the research aim, risks and benefits of participation, and the right to withdraw from the study before data analysis. If interested, they were encouraged to complete a secure online-based survey (hosted by https://www.wjx.cn.). The completion of the questionnaire was considered as giving informed consent to participate in this study. Before data collection, ethical approval was obtained. There were no incentives provided to participants. Inclusion criteria were as follows: (a) aged 18 years old or more; (b) currently enrolled in universities in China with Chinese nationality and (c) have left-behind experience.

The sample size was calculated using the computational formula $N=\frac{{Z}_{\alpha /2}^{2}\times P\times (1-P)}{{\updelta }^{2}}$. We chose* P* = 50%, a 95% confidence level(α) and an allowable error (δ) of 3%. The maximum sample size was 1067.

### Measures

The survey consisted of a self-designed socio-demographic questionnaire based on factors mentioned by United Nations International Children’s Emergency Fund [46] and five validated measures. The socio-demographic included 12 items as follows: grade, gender, place of origin, only child or not, parental marriage, father’s education level, mother’s education level, monthly income, type of absence, age of children being left behind, length of left-behind time, contact frequency with parents. The five validated measures are as follows:

#### The Depression, Anxiety, and Stress Scales-21 (DASS‐21)

DASS‐21 [29] was used to investigate depression, anxiety, and stress, and it has been translated into Chinese [19]. The scale consists of 21 items and three dimensions. The item score ranges from 0 (“did not apply to me at all”) to 3 (“applied to me very much”). A higher score of a dimension indicates higher levels of depression or anxiety or stress. In this study, Cronbach’s alpha was 0.91 for the DASS, 0.86 for both the depression and stress subscale and 0.82 for the anxiety subscale.

#### The Pittsburgh Sleep Quality Index (PSQI)

The Chinese version of PSQI [28] consists of 24 items and seven components: sleep quality, sleep latency, sleep duration, habitual sleep efficiency, sleep disturbances, use of sleep medications, and daytime dysfunction. The item score ranges from 0 to 3, and a higher score indicates poorer subjective sleep quality. Cronbach’s alpha of PSQI was 0.73 in this study.

#### Psychological flexibility

##### (1) Acceptance and Action Questionnaire (AAQ-II)

AAQ-II [16] was used to evaluate experiential avoidance, and it has been translated into Chinese [7]. The scale consists of seven items and the item score ranges from 1 (“Never true”) to 7 (“Always true”). A higher score represents greater experience avoidance. The Cronbach’s alpha was 0.93 in this study.

##### (2) Cognitive Fusion Questionnaire (CFQ)

CFQ [17] was developed to assess cognitive fusion, consisting of seven items and two dimensions: cognitive fusion (CFQ-F) and cognitive defusion (CFQ-D). The Chinese version only contains CFQ-F with seven items [53], and the item score ranges from 1 (“Never true”) to 7 (“Always true”). A higher score represents a higher degree of cognitive fusion and the Cronbach’s alpha of CFQ-F was 0.96 in this study.

##### (3) Valuing Questionnaire (VQ)

VQ [40] was designed to assess overall valued living, we used the Chinese version [5]. The scale consists of 10 items and two dimensions: Progress (VQ-P) and Obstruction (VQ-O). The item score ranges from 0 (“not at all true”) to 6 (“completely true”). A higher VQ-P score indicates a closer alignment between one’s values and actions, while a higher VQ-O score indicates more interference with living consistently with one’s values. In this study, Cronbach’s alpha was 0.88 for VQ-P and 0.81 for VQ-O.

### Data Analysis

Mplus 8.3 [32] and SPSS 26.0 were used for data analysis. Before conducting LPA, we scaled the scores of VQ-P, VQ-O, AAQ-II, and CFQ to facilitate the interpretation of the profiles. Specifically, we converted the raw scores into T-scores. The scores of VQ-O, AAQ-II and CFQ were turned into positive scores so that a higher score of any one indicator presented better PF.

LPA was conducted based on three dimensions of PF (values, acceptance and cognitive defusion). LPA is a model testing process where the best-fitting model is usually selected after comparing 5 to 6 models regarding the following indicators: (1) Information indicators [33]: Akaike Information Criteria (AIC), Bayesian Information Criteria (BIC) and Adjusted Bayesian Information Criterion (aBIC); (2) Classification indicator: Entropy; and (3) Indicators of Likelihood Ratio Test: LO-Mendell-Rubin (LMR) Test and Bootstrapped Likelihood Ratio Test (BLRT). In this study, an optimal model fit was defined: (1) AIC, BIC, and aBIC are approximately the smallest in the model; (2) Entropy > 0.7; (3) LMR and BLRT were significant (P < 0.05). A model would be excluded if there is a small profile containing less than 5% of the sample, as the small profile may be spurious and it may be difficult to represent a distinct profile when generalizing to other groups [15]. We used the ML three-step approach for LPA covariate inclusion [15, 48].

There was no missing data because the questionnaire could only be submitted with the completion of all items. Descriptive analyses (conforming to normality) were conducted using means and standard deviations. ANOVA was used to compare the differences among PF profiles for depression, anxiety, stress, and sleep disorder. χ2 analysis was used to compare socio-demographic variables among different profiles, and adjusted standardized residual (ASR) was used to measure the strength of the difference between observed and expected values, with values higher than 2.0 (or lower than 2.0) indicating the observed frequency of one cell is significantly higher (or lower) than the expected frequency. The relation between multiple variables was presented by logistic regression. The two-sided test was used, and *P* < 0.05 indicates statistical significance in this study.

## Results

### Descriptive Results

A sample of 1988 university students with left-behind experience participated in this study. Table 1 shows the mean and SD (raw scores) of the variables and the correlations between the variables. The values progress was negatively correlated with depression, anxiety, and stress levels and positively correlated with sleep disorder with low correlation coefficients. Experiential avoidance, cognitive fusion and values obstruction were positively correlated with depression, anxiety, stress, and sleep disorder.

**Table 1:** Means, standard deviation, and correlations between variables (N = 1988)

	M	SD	1	2	3	4	5	6	7	8
1. VQ-P	16.50	6.83	1							
2. VQ-O	18.55	6.05	−.18**	1						
3. AAQ-II	38.29	8.04	−.04	.61**						
4. CFQ	43.08	13.15	−.14**	.71**	.81**	1				
5. Depression	1.49	0.90	−.17**	.47**	.59**	.47**	1			
6. Anxiety	1.84	1.18	−.09**	.51**	.66**	.56**	.72**	1		
7. Stress	1.26	0.70	−.07**	.38**	.52**	.43**	.65**	.65**	1	
8. Sleep	4.76	2.88	.03	.41**	.48**	.45*	.38**	.45**	.35**	1

VQ-P = Process of Valuing Questionnaire; VQ-O = Obstruction of Valuing Questionnaire; AAQ-II = Acceptance and Action Questionnaire-II; CFQ = Cognitive Fusion Questionnaire

*p < .05

**p < .01

### Latent-Profile Analysis Results

Table 2 presents the model fit indices for the latent-profile analysis of PF. The results of LPA suggested that a four-profile fits the data best. We excluded the five- and six-profile model because both were smaller profiles with less than 5% of the total participants. The four-profile model had lower values of AIC, BIC, and aBIC (AIC = 55,069.634, BIC = 55,198.316, aBIC = 55,125.244) than the two- and three-profile model. In addition, the entropy of the four-profile model was 0.834 (> 0.7).

**Table 2:** Model fit indices for latent-profile analysis of PF (N = 1988)

Model	K	Log(L)	AIC	BIC	aBIC	Entropy	LMR(*P*)	BLRT(*P*)	CP
1	8	−29,591.555	59,199.111	59,243.870	59,218.453	–	–	–	1
2	13	−28,343.175	56,712.351	56,785.084	56,743.783	.826	< .001	< .001	.532/ .468
3	18	−27,882.699	55,801.399	55,902.107	55,844.920	.858	< .001	< .001	.491/ .102/ .406
4	23	−27,511.817	55,069.634	55,198.316	55,125.244	.834	< .001	< .001	.106/ .248/ .239/ .406
5	28	−27,323.735	54,703.469	54,860.126	54,771.169	.847	< .001	< .001	.216/ .104/ .356/ .045/ .279
6	33	−27,249.689	54,565.379	54,750.010	54,645.167	.823	< .001	< .001	.091/ .091/ .273/ .286/ .216/ .044

*K* = free parameter estimates; *Log(L)*: Log-likelihood ratio test; *AIC* = Akaike information criterion; BIC = Bayesian information criterion; *ABIC* = Adjusted *BIC*; *LMR* = Lo-Mendell-Rubin; *BLRT* = Bootstrap likelihood ratio test; *CP* = category probability

**Fig. 1:**
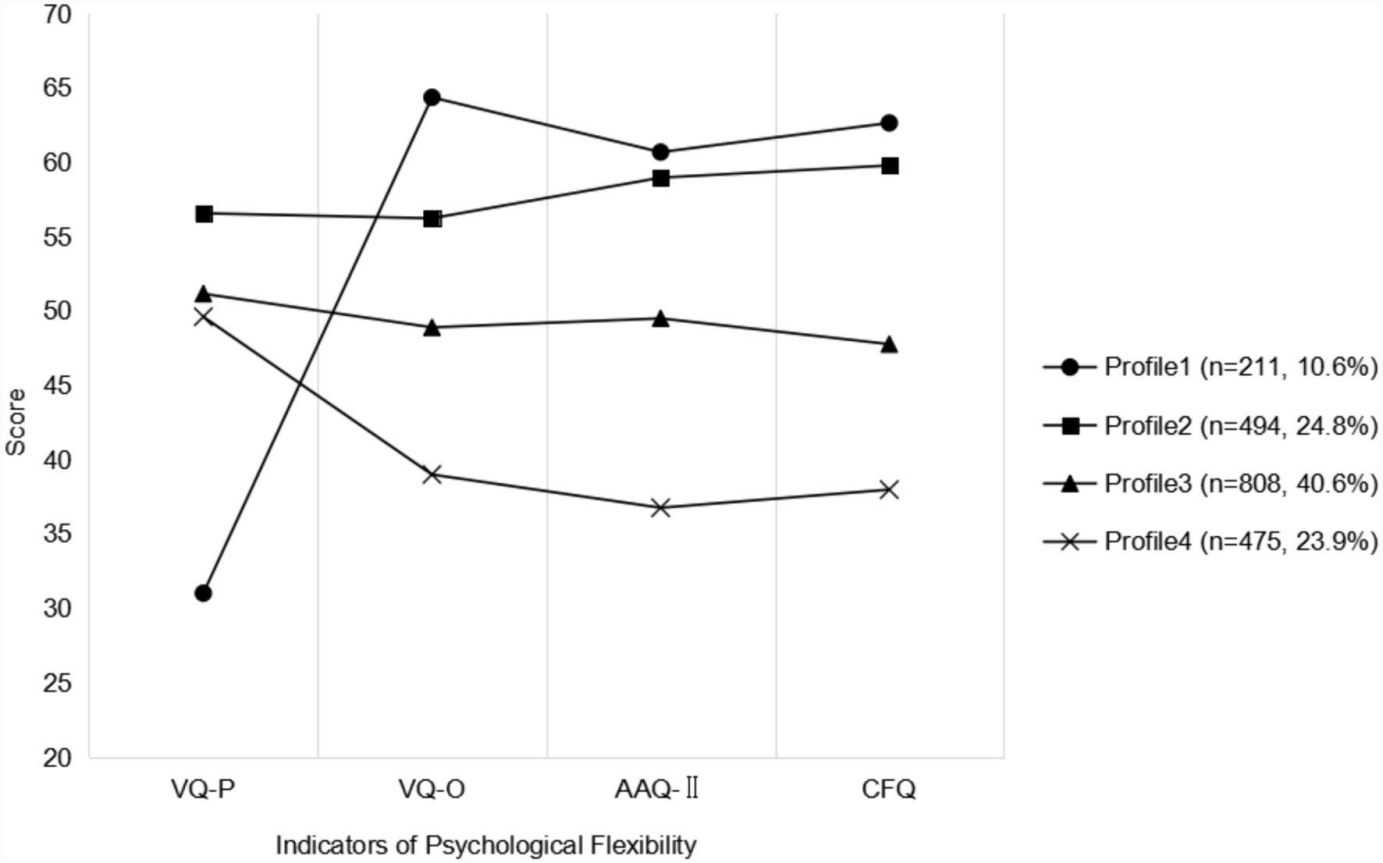
Parameter estimates for the four-profile model. *Note.* (1) VQ-P = Process of Valuing Questionnaire; (2) VQ-O = Obstruction of Valuing Questionnaire; AAQ-II = Acceptance and Action Questionnaire-II; CFQ = Cognitive Fusion Questionnaire. (3) Profile 1 = somewhat flexible& low value progress, Profile 2 = highly flexible, Profile 3 = moderately flexible, Profile 4 = low flexible

Parameter estimates for the four-profile model are shown in Table 3 and Fig. 1**,** and the converted positive score was used in subsequent analysis. We labeled the first profile as “somewhat flexible& low values progress” (profile 1, n = 211, 10.6%), in which participants reported the lowest VQ-P score and the highest scores in VQ-O, AAQ-II and CFQ. The second profile was described as “highly flexible” (profile 2, n = 494, 24.8%) with high scores on all domains. The third profile was labeled as “moderately flexible” (profile 3, n = 808, 40.6%), with moderate scores on all domains. We labeled the last profile as “low flexible” (profile 4, n = 475, 23.9%) because participants in this profile had a low score of VQ-P, VQ-O, AAQ-II and CFQ.

**Table 3:** The score of VQ-P, VQ-O, AAQ-II and CFQ among four profiles

	Profile 1	Profile 2	Profile 3	Profile 4	F	*P*
VQ-P	31.01 ± 4.98	56.57 ± 6.93	51.16 ± 7.76	49.61 ± 6.92	651.29	< .001***
VQ-O	64.37 ± 4.43	56.23 ± 6.98	48.90 ± 6.27	39.00 ± 6.09	1035.70	< .001***
AAQ-II	60.69 ± 4.19	58.97 ± 3.97	49.50 ± 5.23	36.76 ± 6.14	1895.84	< .001***
CFQ	62.65 ± 3.51	59.80 ± 4.27	36.76 ± 6.14	37.99 ± 5.37	2264.02	< .001***

(1) VQ-P = Process of Valuing Questionnaire; (2) VQ-O = Obstruction of Valuing Questionnaire; AAQ-II = Acceptance and Action Questionnaire-II; CFQ = Cognitive Fusion Questionnaire. (3) Profile 1 = somewhat flexible& low value progress, Profile 2 = highly flexible, Profile 3 = moderately flexible, Profile 4 = low flexible

***p < .001

### Socio-Demography and Left-Behind Related Characteristics of Participants Regarding PF Profiles

As shown in Table 4, undergraduates made up the most participants across four profiles. 73.0% of the participants came from the rural areas. As for left-behind experience, 65.3% of students experienced two parents’ absence; 62.5% of students were left behind since kindergarten and 29.1% since primary school; the majority being left behind less than 5 years; and most students contact with parents more often than once a month.

**Table 4:** Socio-demography and left-behind related characteristics of participants regarding PF profiles (N = 1988)

Demographics		Latent Profile N (%)				χ2	*P*
		Profile 1	Profile 2	Profile 3	Profile 4		
Grade	Undergraduate	180 (85.3)	423 (85.6)	754 (93.3)	430 (90.5)	25.401	< .001***
	Graduate	31 (14.7)	71 (14.4)	54 (6.7)	45 (9.5)		
Gender	Males	93 (44.1)	152 (30.8)	202 (25.0)	94 (19.8)	48.658	< .001***
	Females	118 (55.9)	342 (69.2)	606 (75.0)	381 (80.2)		
Place of origin	Township	188(89.1)	445 (90.1)	724 (89.6)	416 (87.6)	1.830	.608
	City	23 (10.9)	49 (9.9)	84 (10.4)	59 (12.4)		
Only child or not	Yes	30 (14.2)	63 (12.8)	117 (14.5)	71 (14.9)	1.116	.773
	No	181 (85.8)	431 (87.2)	691 (85.5)	404 (85.1)		
Parental marriage	Married	183 (86.7)	426 (86.2)	687 (85.0)	387 (81.5)	5.435	.143
	Others: divorce, bereaved spouse, remarriage and separated	28 (13.3)	68 (13.8)	121 (15.0)	88 (18.5)		
Father’s education level	Primary school	57 (27.0)	108 (21.9)	197 (24.4)	124 (26.1)	3.249	.355
	Middle school	154 (73.0)	386 (78.1)	611 (75.6)	351 (73.9)		
Mother’s education level	Primary school	87 (41.2)	218 (44.1)	378 (46.8)	204 (42.9)	3.095	.377
	Middle school	124 (58.8)	276 (55.9)	430 (53.2)	271 (57.1)		
Monthly income	< 3 k	136 (64.5)	325 (65.8)	512 (63.4)	304 (64.1)	2.405	.879
	3-8 k	63 (29.9)	146 (29.6)	262 (32.4)	152 (32.0)		
	> 10 k	12 (5.7)	23 (4.7)	34 (4.2)	19 (4.0)		
Experience about left-behind							
Type of absence	Father absence	61 (28.9)	114 (23.1)	202 (25.0)	123 (25.9)	4.491	.876
	Mother absence	11 (5.2)	29 (5.9)	45 (5.6)	21 (4.4)		
	Rotating absence	7 (3.3)	19 (3.8)	36 (4.5)	21 (4.4)		
	Two parents’ absence	132 (62.6)	332 (67.2)	525 (65.0)	310 (65.3)		
Age of children being left behind	Kindergarten or earlier	133 (63.0)	297 (60.1)	493 (61.0)	319 (67.2)	18.873	.004**
	Primary school	62 (29.4)	147 (29.8)	238 (29.5)	132 (27.8)		
	Middle school	16 (7.6)	50 (10.1)	77 (9.5)	24 (5.1)		
Length of left-behind time	≤ 5y	169 (80.1)	328 (66.4)	546 (67.6)	288 (60.6)	25.258	< .001***
	> 5y	42 (6.4)	166 (33.6)	262 (32.4)	187 (39.4)		
Contact frequency with parents	More often than once a month	164 (77.7)	406 (82.2)	617 (76.4)	343 (72.2)	25.500	< .001***
	Once within 1–6 months	28 (13.3)	63 (12.8)	126 (15.6)	68 (14.3)		
	Once every 6 months or more	19 (9.0)	25 (5.1)	65 (8.0)	64 (13.5)		

Profile 1 = somewhat flexible& low value progress, Profile 2 = highly flexible, Profile 3 = moderately flexible, Profile 4 = low flexible

**p < .01

***p < .001

The distribution of grade, gender, length of left-behind time and contact frequency with parents were significantly different among profiles (*P* < 0 0.05). Participants who are graduates, males and being left behind within 5 years were overrepresented in profile “somewhat flexible & low values progress” (ASR = 2.3, 5.8, 4.3, respectively). As for profile “highly flexible”, participants who are graduates and contacting with parents once within 1 month were overrepresented in this profile (ASR = 3.6 and 3.2, respectively). Participants who are females, with other parental marriage, being left behind since kindergarten or earlier, been left behind over 5 years and contacting with parents once every 6 months or more were overrepresented in profile “low flexible” (ASR = 4.2, 2.2, 2.4, 3.4 and 4.2 respectively).

### Differences Between Psychological Flexibility Profiles for Depression, Anxiety, Stress and Sleep

**Fig. 2:**
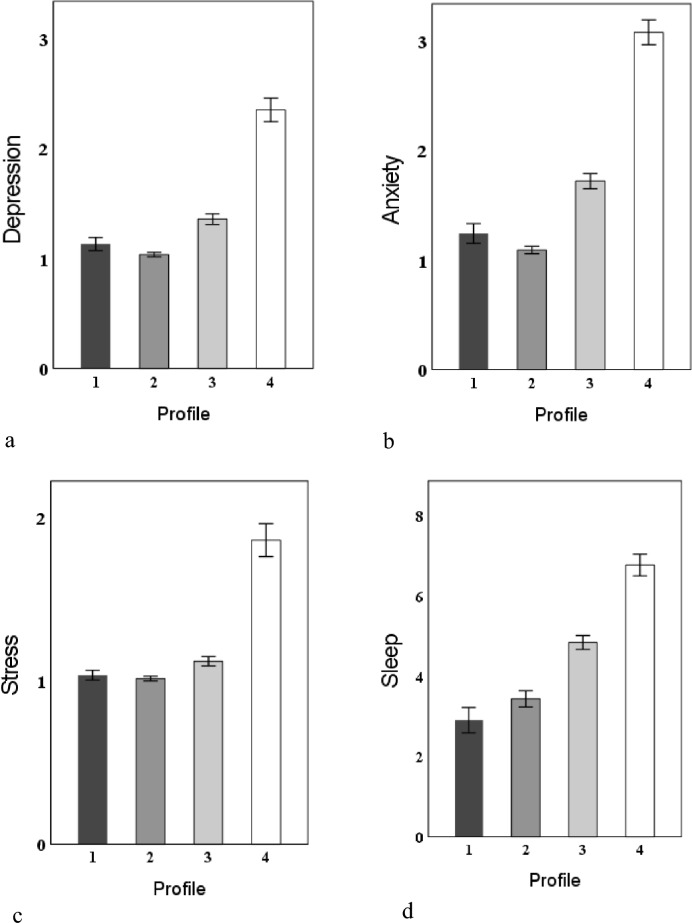
The scores of depression, anxiety, stress and sleep across the four profiles. *Note.* Profile 1 = somewhat flexible& low value progress, Profile 2 = highly flexible, Profile 3 = moderately flexible, Profile 4 = low flexible

As presented Fig. 2, the score of depression, anxiety, stress and sleep were significantly different across the four profiles (*P* < 0.05). Participants in Profile 2 showed the lowest levels of depression (1.04 ± 0.24), anxiety (1.09 ± 0.37), and stress (1.01 ± 0.17). Participants in Profile 4 showed the highest levels of depression (2.35 ± 1.18), anxiety (3.08 ± 1.24), stress (1.86 ± 1.12), and sleep disorder (6.78 ± 2.98).

### The Association Between Psychological Flexibility Profiles, Social-Demographic Variables, and Experience About Left-Behind

Profile 2 “highly flexible” is the reference group in the multinomial logistic regression analyses. Compared with those in profile “highly flexible”, participants who were females (OR = 0.577, *P* = 0.002) and being left behind over 5 years (OR = 0.473, *P* < 0.001) were less likely to be in profile “somewhat flexible& low values progress”. Graduates (OR = 0.379, *P* < 0.001) were less likely to be in the profile “moderately flexible” compare with those in profile “highly flexible”. Compared with those in profile “highly flexible”, participants who are females (OR = 1.919, *P* < 0.001) and contact with parents once within one to 6 months (OR = 3.084, *P* < 0.001) were more likely to be in profile “low flexible”. Participants who were graduates (OR = 0.542, P = 0.004), being left behind since primary school (OR = 0.517, P = 0.015) were less likely to be in the profile “low flexible” compared with those in profile “highly flexible”. The detail results of multinomial logistic regression are shown in the Supplementary materials (Supplementary Table 1).

## Discussion

The current study aimed to identify PF profiles of university students with left-behind experience, using latent profile analysis and examine associations between profiles and demographic characteristics and mental health (i.e., depression, anxiety, stress, and sleep disorder). Our study found four distinct profiles of PF, which could be influenced by left-behind experience.

In our study, the largest profile of PF is moderately flexibility (n = 808, 40.6%), consistent with a previous study [4] about PF of university students. Besides, 24.8% of participants were characterized as highly flexible, suggesting that the majority of university students with left-behind experience were able to choose a valued life, with a flexible focus on the present moment and behaviors that were not limited by their thinking. However, the proportion of low PF was higher among university students left-behind than among general university students [4]. Exposure to left-behind experience may decrease psychological flexibility. Our study identified a particular profile “somewhat flexible & low values progress”, which has not been found in other studies. Participants in this profile have difficulty in choosing a valued life and may not find the important direction and put it into action. Possibly due to the lack of parental value guidance while growing up, there are some difficulties in choosing a valued life in their adulthood [38].

Our study found that PF was positively correlated with the mental health of university students with left-behind experience, consistent with the results among general university students [4]. Those who had a left-behind experience lacked someone to talk to, maybe less able to dissipate negative feelings, and tend to avoid negative emotions and experiences. However, if people had a moderate or high level of PF, they were able to accept their thoughts and feelings and choose live according to their values. Previous studies also found that higher values progress and lower values obstruction, experiential avoidance and cognitive fusion predict better mental health [3, 40]. However, students in profile “somewhat flexible& low values progress” have low levels of depression, anxiety and stress, and the best sleep quality. One possible reason is that values progress is more related to life satisfaction and purpose, rather than to mental health problems; whereas values obstruction is more related to negative moods [40]. As a result, individuals in profile “somewhat flexible& low values progress” had good mental health despite the lowest level of values progress.

Consistent with the findings of a previous study on the PF of university students [4], we found that female university students with left-behind experience showed a relatively large distribution in the “low flexible” profile. One possible reason is that females tend to cope with negative emotions by rumination compared to males, i.e., thinking repeatedly and unproductively about their negative emotions [23]. Rumination has been conceptualized as an avoidance strategy and is associated with experiential avoidance [18]. Another possible reason is that girls’ emotional and material needs are more often neglected than boys’ needs, as boys receive more attention than girls in traditional families of some developing countries [46]. Likewise, girls do not receive enough attention to their PF and mental health, making them more likely to characterize by low PF and develop mental disorders as adults.

In our study, participants who started left behind at a young age (kindergarten or earlier), left behind for a long time, and had less frequent contact with their parents were prone to low PF and poorer mental health than others. Students with left-behind experience had more negative events during the growth process [20] than their peers, and they usually have to cope with negative events alone. When their abilities were not sufficient to solve problems, they were more likely to adopt an avoidance attitude toward negative events rather than accepting and actively coping. According to attachment theory [1], childhood (especially 2–3 years old) is a critical period for building attachment relationships with parents. Separation from parents and disruptions in communication can easily lead children to develop insecure attachments, which causes more mental and behavioral problems [37]. In contrast, frequent communication between parents and their children (e.g., communication about feelings, life difficulties, and academic performance) significantly promote parent–child relationship, increase happiness and reduce adverse effects among students with left-behind experience [44, 54]. These implied that students separated from their parents early and for a long time needed more attention and preventive psychological interventions.

In our study, place of origin was not associated with PF profiles and mental health. However, previous studies have found an association between place of origin with mental health and PF, but no consistent conclusions have been reached. One possible reason for no association in this study was that the cultural gap between the township and urban areas has gradually narrowed due to the popularity of the Internet and the economic development of township areas. The influence of the growing environment on left-behind university students had become smaller. In addition, township and urban differences may affect young people’s mental health through neighborhood deprivation, which works by influencing family and peer contexts [49]. For the left-behind university students in the township and urban areas, both grew up in the absence of their parents, so there is little difference in place of origin.

In our study, one-quarter of university students with left-behind experience had low PF and poor mental health. While the role of PF in this group was often neglected. The strength of this study is the use of LPA to distinguish different subgroups of PF, which focused on core components, rather than considered PF as a single structure. LPA helps to determine key components of PF associated with the mental health of left-behind university students, to identify the mechanisms by which PF affects mental health, and to take specific interventions to improve mental health of this group. Given that PF is considered an ability that can be improved, interventions based on Acceptance Commitment Therapy (ACT) can be used to improve PF in individuals with low PF [50] and help improve their mental health. For example, therapists can use ACT to increase PF of those in the “somewhat flexible & low values progress” group by focusing on the level of values progress. Helping individuals to choose a fulfilling and meaningful life is one of the primary goals of ACT, which works through values progress [12]. Participants who are females, have been left behind since kindergarten or earlier, have been left be identify those more likely to be low PF group and take specific intervention for mental health problems based on the PF components. Universities can conduct a mapping survey of students with left-behind experience to identify those more likely to be low PF group and take specific intervention for mental health problems based on the PF components.

The study has several limitations. Firstly, the AAQ-II and CFQ indirectly reflect levels of psychological flexibility by assessing psychological inflexibility, the decrease in “inflexibility” does not fully represent “flexibility” [31]. Secondly, the left-behind experience was obtained through self-reports, which may be subject to recall bias. Finally, the higher education level of left-behind university students requires caution in generalizing the results of this study to other adults with left-behind experience.

## Conclusion

This is the first study using latent profile analysis to explore PF of university students with left-behind experience, although the relation between mental health and PF was consistent with previous studies, the present study provides unique evidence of the impact of the left-behind experience on PF and mental health. Left-behind university students who are females, have been left behind since kindergarten or earlier, have been left behind over 5 years, and contacted parents less than once a month may be more characterized by low PF. More support or interventions are needed to improve their PF and mental health, such as ACT, increasing parent–child interaction during childhood, etc. Longitudinal studies continuously monitoring the mental health and PF from adolescence to early adulthood or trials of the effectiveness of ACT among university students can be conducted in the future, to further explore the role of PF in the mental health of university students with left-behind experience.

## Summary

Left-behind experience has been proven to be a risk factor for mental health of university students, and the number of left-behind children continues to grow. The relation between psychological flexibility and mental health of those with left-behind experience is hardly ever studied. The current study used the latent profile analysis to identify the psychological flexibility subgroups and analyzed the relation between these subgroups and mental health problems. The results show four profiles, among which “moderately flexible” is the largest profile. We also found a special profile “somewhat flexible & low values progress”, and participants in this profile have low levels of mental health problems despite low values progress. Consistent with previous studies, the level of psychological flexibility is positively related to mental health in our study. University students who are females, have been left behind since kindergarten or earlier, have been left behind over 5 years, and contacted parents less than once a month may be more likely to have low psychological flexibility. More attention and scientific interventions are needed to improve psychological flexibility and mental health of university students with these characteristics.

## Supplementary Information

Below is the link to the electronic supplementary material.

Supplementary file1 (DOCX 18 KB)

## Data Availability

The data that support the findings of this study are available from the corresponding author upon reasonable request.
