# Supplementary material for: Psychological Flexibility Profiles and Mental Health Among University Students with Left-Behind Experience: A Latent Profile Analysis
Source: Child Psychiatry Hum Dev. 2024 Jun 12;57(2):456–66. doi: 10.1007/s10578-024-01720-3 (PMC13128777; doi:10.1007/s10578-024-01720-3)
Supplement: Supplementary file 1 — Supplementary file1 (DOCX 18 KB) [file 10578_2024_1720_MOESM1_ESM.docx]

**Supplementary Table 1**

Logistic regression analysis of profiles of PF of university students with left behind experience (N = 1988).

|  | **B** | **Odds ratio** | ***p*** |
| --- | --- | --- | --- |
| **Profile 1 (vs. Profile 2)** |  |  |  |
| Grade: graduate, ref.: undergraduate | 0.088 | 1.092 | .716 |
| Gender: females, ref.: males | -0.549 | 0.577 | .002** |
| Place of origin: city, ref.: township | 0.007 | 1.007 | .981 |
| Only child or not: no, ref.: yes | 0.043 | 1.044 | .871 |
| Parental marriage: others^1^, ref.: married | -0.064 | 0.938 | .795 |
| Father's education level: middle school, ref.: primary school | -0.371 | 0.690 | .071 |
| Mother's education level: middle school, ref.: primary school | 0.206 | 1.229 | .271 |
| Monthly income: 3~8k, ref.: <3k | 0.217 | 1.243 | .578 |
| Monthly income: >10k, ref.: <3k | 0.049 | 1.051 | .799 |
| Type of absence: mother absence, ref.: father absence | -0.201 | 0.818 | .309 |
| Type of absence: rotating absence, ref.: father absence | -0.432 | 0.649 | .363 |
| Type of absence: two parents  absence, ref.: father absence | -0.267 | 0.765 | .498 |
| Age of children being left behind: primary school, ref.: kindergarten or earlier | -0.500 | 0.607 | .112 |
| Age of children being left behind: middle school, ref.: kindergarten or earlier | -0.177 | 0.838 | .350 |
| Length of left-behind time: >5y, ref.: ≤5y | -0.748 | 0.473 | <.001*** |
| Contact frequency with parents: once within 1~6 months, ref.: more often than once a month | 0.627 | 1.871 | .054 |
| Contact frequency with parents: once every 6  months or more, ref.: more often than once a month | 0.086 | 1.089 | .731 |
| **Profile 3 (vs. Profile 2)** |  |  |  |
| Grade: graduate, ref.: undergraduate | 0.197 | 0.379 | <.001*** |
| Gender: females, ref.: males | 0.130 | 1.425 | .007** |
| Place of origin: city, ref.: township | 0.207 | 1.016 | .940 |
| Only child or not: no, ref.: yes | 0.185 | 0.774 | .166 |
| Parental marriage: others^1^, ref.: married | 0.168 | 1.061 | .724 |
| Father's education level: middle school, ref.: primary school | 0.146 | 0.889 | .419 |
| Mother's education level: middle school, ref.: primary school | 0.128 | 0.846 | .193 |
| Monthly income: 3~8k, ref.: <3k | 0.293 | 1.049 | .871 |
| Monthly income: >10k, ref.: <3k | 0.133 | 1.244 | .102 |
| Type of absence: mother absence, ref.: father absence | 0.143 | 0.841 | .226 |
| Type of absence: rotating absence, ref.: father absence | 0.312 | 1.066 | .839 |
| Type of absence: two parents  absence, ref.: father absence | 0.271 | 0.846 | .536 |
| Age of children being left behind: primary school, ref.: kindergarten or earlier | 0.204 | 0.958 | .833 |
| Age of children being left behind: middle school, ref.: kindergarten or earlier | 0.133 | 1.047 | .730 |
| Length of left-behind time: >5y, ref.: ≤5y | 0.127 | 0.973 | .826 |
| Contact frequency with parents: once within 1~6 months, ref.: more often than once a month | 0.248 | 1.859 | .012* |
| Contact frequency with parents: once every 6  months or more, ref.: more often than once a month | 0.170 | 1.432 | .035* |
| **Profile 4 (vs. Profile 2)** |  |  |  |
| Grade: graduate, ref.: undergraduate | 0.212 | 0.542 | .004** |
| Gender: females, ref.: males | 0.156 | 1.919 | <.001*** |
| Place of origin: city, ref.: township | 0.226 | 1.278 | .278 |
| Only child or not: no, ref.: yes | 0.208 | 0.815 | .325 |
| Parental marriage: others^1^, ref.: married | 0.183 | 1.357 | .095 |
| Father's education level: middle school, ref.: primary school | 0.163 | 0.757 | .089 |
| Mother's education level: middle school, ref.: primary school | 0.145 | 1.029 | .843 |
| Monthly income: 3~8k, ref.: <3k | 0.337 | 0.875 | .691 |
| Monthly income: >10k, ref.: <3k | 0.151 | 1.163 | .317 |
| Type of absence: mother absence, ref.: father absence | 0.160 | 0.755 | .080 |
| Type of absence: rotating absence, ref.: father absence | 0.352 | 1.140 | .709 |
| Type of absence: two parents  absence, ref.: father absence | 0.323 | 0.602 | .117 |
| Age of children being left behind: primary school, ref.: kindergarten or earlier | 0.271 | 0.517 | .015* |
| Age of children being left behind: middle school, ref.: kindergarten or earlier | 0.151 | 0.948 | .724 |
| Length of left-behind time: >5y, ref.: ≤5y | 0.141 | 1.233 | .137 |
| Contact frequency with parents: once within 1~6 months, ref.: more often than once a month | 0.252 | 3.084 | <.001*** |
| Contact frequency with parents: once every 6  months or more, ref.: more often than once a month | 0.193 | 1.321 | .149 |

*Note.* (1) Profile 1 = somewhat flexible& low value progress, Profile 2 = highly flexible, Profile 3 = moderately flexible, Profile 4 = low flexible. (2) ^1^Others include divorce, bereaved spouse, remarriage and separated. (3) All statistical tests are two-sided. **p* < .05, ***p* < .01, ****p* < .001. OR (odds ratio) and 95% CI (95% confidence interval) were calculated using binary logistic regression.
